# Therapeutic strategies in gastroparesis: Results of stepwise approach with diet and prokinetics, Gastric Rest, and PEG‐J: A retrospective analysis

**DOI:** 10.1111/nmo.13588

**Published:** 2019-04-04

**Authors:** Denise Strijbos, Daniel Keszthelyi, Fabiënne G. M. Smeets, Joanna Kruimel, Lennard P. L. Gilissen, Rogier de Ridder, José M. Conchillo, Ad A. M. Masclee

**Affiliations:** ^1^ Division of Gastroenterology and Hepatology, Department of Internal Medicine, NUTRIM School of Nutrition and Translational Research in Metabolism Maastricht University Medical Centre Maastricht The Netherlands; ^2^ Department of Gastroenterology and Hepatology Catharina Hospital Eindhoven Eindhoven The Netherlands

**Keywords:** enteral nutrition, gastroparesis, percutaneous endoscopic gastrostomy

## Abstract

**Background:**

Gastroparesis is characterized by abnormal gastric motor function with delayed gastric emptying in the absence of mechanical obstruction. In our tertiary referral center, patients are treated with a stepwise approach, starting with dietary advice and prokinetics, followed by three months of nasoduodenal tube feeding with “gastric rest.” When not successful, a percutaneous endoscopic gastrostomy with jejunal extension (PEG‐J) for long‐term enteral feeding is placed.

**Aim:**

To evaluate the effect of this stepwise approach on weight and symptoms.

**Methods:**

Analyses of data of all referred gastroparesis patients between 2008 and 2016.

**Key Results:**

A total of 86 patients (71% female, 20‐87 years [mean 55.8 years]) were analyzed of whom 50 (58%) had adequate symptom responses to diet and prokinetics. The remaining 36 (decompensated gastroparesis) were treated with three months gastric rest. Symptom response rate was 47% (17/36). Significant weight gain was seen in all patients, independent of symptom response. In the remaining 19 symptom non‐responders, the enteral feeding was continued through PEG‐J. Treatment was effective (symptoms) in 37%, with significant weight gain in all. In 84% of patients, the PEG‐J is still in use (mean duration 962 days).

**Conclusions and Inferences:**

Following a stepwise treatment approach in gastroparesis, adequate symptom response was reached in 86% of all patients. Weight gain was achieved in all patients, independent of symptom response. Diet and prokinetics were effective with regard to symptoms in 58%, temporary gastric rest in 47%, and PEG‐J as third step in 37% of patients.

## Graphical abstract

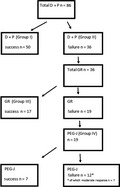

**Key points**

- Symptom management of gastroparesis is challenging, due to an incomplete understanding and limited therapeutic options. About 30% of patients eventually need enteral feeding.
- Following a stepwise treatment approach (including diet and prokinetics, Gastric Rest, PEG‐J) in gastroparesis, adequate symptom response was reached in 86% of all patients, whereas weight gain was achieved in all patients, independent of symptom response.
- The burden of this disorder on individuals and society is considerable and might be relieved by a stepwise approach.

## 1 INTRODUCTION

Gastroparesis is a chronic disorder of the stomach characterized by delayed gastric emptying, in the absence of mechanical obstruction. Symptoms of gastroparesis include nausea, satiation, postprandial fullness, vomiting, upper abdominal pain, and bloating.^1^, ^2^, ^3^ The estimated age‐adjusted incidence of gastroparesis in 2007 was 37.8 per 100 000 persons in women, and 9.6 per 100 000 in men.^4^ The burden of this disorder on individuals (morbidity, quality of life, and mortality) and society (health care costs and indirect costs through productivity loss) is considerable.^4^, ^5^

Although gastroparesis is frequently associated with diabetes (diabetic gastroparesis, DGP), idiopathic gastroparesis (IGP) accounts for the majority of cases (60%). Other known causes of gastroparesis are surgery (eg, bariatric, antireflux), related to vagotomy, medications, metabolic derangements, or following bacterial or viral infections.^6^, ^7^ The pathophysiology of gastroparesis is incompletely understood. Several mechanisms have been postulated including impaired gastric accommodation, antral hypomotility, dysregulation of antroduodenal coordination,^8^ pyloric dysfunction, and excessive inhibitory duodenogastric feedback.^9^, ^10^, ^11^

Gastroparesis can be classified into the following categories: grade 1 (easily controlled symptoms, ability to maintain weight on regular or slightly adjusted diet); grade 2 is compensated gastroparesis (moderate symptoms, partial control by medication, ability to maintain nutritional status with dietary adjustments, and occasional hospital admissions). Grade 3 is decompensated gastroparesis (refractory symptoms, nutritional status cannot be maintained via oral intake, and frequent hospital admissions 2, 12).

Due to an incomplete understanding of gastroparesis and limited therapeutic options, symptom management is challenging. Nutritional interventions are often necessary. Initial treatment generally consists of dietary interventions (eg, intake of small and frequent meals) and restoring glycemic control in diabetic patients.^13^, ^14^ Dietary advices are often combined with use of prokinetic agents such as dopaminergic‐2‐antagonists (eg, domperidone, metoclopramide), motilin‐analogues (eg, erythromycin), or serotonin_4_ (5‐HT_4_)‐agonists (eg, prucalopride). However, lack of robust evidence for their effectiveness in addition to potential serious side effects (QT‐prolongation) or tachyphylaxis limit the chances for a therapeutic benefit.^15^, ^16^, ^17^ With regard to nutritional support, consensus has been reached that nasoduodenal tube feeding should be considered in case patients have significant weight loss (5%‐10% in, respectively, 3‐6 months), are unable to achieve their target weight, or when repeated hospital admissions for malnutrition or dehydration are necessary.^3^, ^18^, ^19^

In our tertiary referral center (Maastricht University Medical Centre, Maastricht UMC+), we provide medical and nutritional support according to a stepwise approach, starting with dietary and lifestyle advice, and prokinetics. When these initial measures fail, in the presence of malnutrition (grade 3 gastroparesis), three months nasoduodenal tube feeding with the aim to achieve “gastric rest” (GR) and “gastric decompression” is considered. Thereafter oral feeding is re‐introduced, in a stepwise fashion and nasoduodenal feeding is stopped. When oral reintroduction fails, placement of a percutaneous endoscopic gastrostomy with jejunal extension (PEG‐J) for long‐term enteral nutrition is offered.

Aim of the present study was to evaluate the efficacy of our stepwise approach with different interventions (diet and medication; nasoduodenal tube feeding with gastric rest; PEG‐J) on gastroparesis symptoms and objective parameters (bodyweight/BMI).

## 2 METHODS

### 2.1 Patients

Records of all referred gastroparesis patients seen between 2008 and 2016 in the Maastricht University Medical Centre (Maastricht UMC+), a tertiary referral center, were reviewed. Patients were identified by reviewing gastric scintigraphy test databases (n = 737) and all PEG‐J placement (n = 496) and DRG codes for gastroparesis (n = 99). Patients with documented evidence for delayed gastric emptying of solids by scintigraphy, exclusion of mechanic obstruction by gastroscopy, and symptoms of gastroparesis (defined as nausea, early satiety, postprandial fullness, vomiting, upper abdominal pain, and bloating) were included in the retrospective analysis. Medical history was reviewed to assess response to treatment according to our stepwise approach. Data assessment included results of gastric emptying tests and antroduodenal manometry. The local ethical committee gave approval to contact patients for additional information. In case of insufficient data, for example in case of loss to follow‐up, patients were excluded.

### 2.2 Diagnostics

#### 2.2.1 Scintigraphy

In the Maastricht University Medical Center (MUMC+), scintigraphy was performed according to standard procedures.^20^, ^21^ Medication affecting gastrointestinal tract motility was stopped at least 3 days prior to the study. All participants underwent scintigraphy after an overnight fast.

A standard meal was given to the patient, consisting of 100 ml egg whites mixed with 99 mTc nanocolloïd (microwaved for 2 minutes), one slice of bread with 15 g jam, and 120 ml radiolabeled (111 In DTPA) water. Patients were instructed to finish this meal within 10 min. Immediately after meal ingestion, images were obtained (t = 0) as well as 60, 120, and 240 minutes after the meal.

Six patients were diagnosed with gastroparesis by a short scintigraphy test (90‐120 minutes), and one patient was diagnosed by a 13C‐octanoic acid breath test. As comparison of different tests and/or study protocols is not possible, we only report quantitative results of the recommended four‐hour solid‐phase gastric emptying scintigraphy. For the 4‐hour solid‐phase gastric emptying scintigraphy, total gastric counts were normalized to 100% for t = 0 (0 minutes; image directly after meal ingestion). The percentage of contents remaining in the stomach was reported after 60, 120, and 240 minutes. Normal values were 53%‐79% for 60 minutes, 16%‐37% for 120 minutes, and 0%‐4% for 240 minutes, as in the validated standard protocol used at our hospital. Normal values for solid and liquid gastric half emptying time were 64‐103 minutes and < 22 minutes, respectively.

#### 2.2.2 High‐resolution antroduodenal manometry

Subjects underwent antroduodenal manometry (ADM) after an overnight fast of at least 8 hours following standard procedures.^22^, ^23^, ^24^, ^25^ Medication affecting gastrointestinal tract motility was stopped 3 days prior to the study. A solid‐state high‐resolution catheter consisting of 36 transducers was used (Unisensor AG, Attikon, Switzerland). Transducers were spaced at 1‐cm intervals. After 30 minutes recording in fasting conditions, subjects were given a standardized meal consisting of one scrambled egg, two slices of white bread with 5 mg of margarine, and 150 ml of water (283 kcal, 41.2% fat, 17.1% carbohydrates, 41.6% protein). After ingestion of the meal, data were recorded for 6 hours, during which patients were not allowed to eat or drink.

During the postprandial period, we determined frequency of valid contractions (contractions per min), contraction amplitude (mm Hg), and motility index of antral pressure waves (mm Hg). Motility index (MI) was calculated using the formula: MI = ln ([number of waves × Σ amplitude] +1) in the two pressure sensors located proximally to the manometrically and fluoroscopically defined pylorus, as previously described. Antral hypomotility was considered as a postprandial antral motility index < 13.67 mm Hg.^26^

All patients proceeding to PEG‐J underwent antroduodenal manometry, except for two patients (in one, the ADM could not be finished due to vomiting, and in a second patient in which case a generalized motility disorder was highly suspected based on medical history, ie, mitochondrial myopathy).

### 2.3 Treatments

#### 2.3.1 Diet and prokinetic treatment (DP)

Dietary advice consisted of a low‐fat, low‐fiber diet, with frequent small meals, prescribed by a dedicated nutritionist which is based on recommendations formulated previously.^16^, ^27^, ^28^ Prokinetics (erythromycin, metoclopramide, or domperidone) were prescribed in case dietary advice was insufficient.

Our first choice for chronic medication is erythromycin (250 mg tid), for this is most effective in improving gastric emptying and improvement of symptom scores.^29^ The major issue with erythromycin is tachyphylaxis due to downregulation of the motilin receptor^17^ in addition to growing concerns surrounding bacterial resistance and the need for appropriate antibiotic stewardship. Therefore, we rotated treatment cycles, with a maximum period of 4 consecutive weeks of medication with one‐week “rest.” Erythromycin was combined with nutritional support from a dietician. In case patients did not respond to erythromycin, we prescribed domperidone or metoclopramide for 4‐week periods (both 10 mg tid). Patients who experienced more nausea compared to early satiety or postprandial fullness as cardinal symptom generally received metoclopramide, otherwise domperidone. The main issue with these dopaminergic‐2‐antagonists is its side effects: metoclopramide may evoke tardive dyskinesia, QT‐prolongation, and depression, whereas domperidone can cause QT‐prolongation.^2^, ^30^ QT intervals were checked routinely prior to treatment only in case there was a history of cardiac disease, when 50 years or older of age or other in case co‐medication was used with a potential to prolong QT intervals. Despite safety concerns, a recent meta‐analysis 31 and review 32 stated that cardiac arrhythmias or adverse events are rare.

Prucalopride was not subscribed as this drug is not reimbursed in the Netherlands.

#### 2.3.2 Gastric Rest (GR)

A nasoduodenal tube was placed endoscopically. Thereafter, complete nasoduodenal tube feeding was given for three months (Gastric Rest), with nil per mouth.

#### 2.3.3 PEG‐J

A PEG was placed according to the standard pull‐method by Gauderer et al,^33^ with immediate placement of a jejunal extension tube through the PEG tube, which was placed in the proximal jejunum and fixed with an hemoclip.^34^, ^35^

### 2.4 Outcome parameters

Of primary interest was the effect of the interventions (diet and prokinetics; intraduodenal tube feeding with gastric rest; PEG‐J) on subjective treatment response, determined according to the Global Physician Assessment Scale 36, 37 (GPA 1: complete relief; GPA 2: marked relief; GPA 3: moderate relief; GPA 4: slight relief; GPA 5: no relief; GPA 6: worsening symptoms), based on the clinical effect reported by the patient during visits to the outpatient department. GPA score was documented four weeks after onset of treatment. Reported complete and marked relief (GPA 1‐2) was considered as a successful treatment response (responders). In case treatment was considered not successful (based on repeated symptom scores), patients were classified as non‐responders and were offered the next step of treatment. Preferably, each medication was tested for at least a four‐week period. Gastric rest with nasoduodenal feeding was given for 3 months. PEG‐J treatment was evaluated regularly, with objective outcomes measured after six months.

Other parameters assessed included the effect on bodyweight (BMI). In this respect, interruption of weight loss or an increase in weight were considered as treatment success, whereas persistent weight loss was considered as treatment failure.

Success rates of the treatments, occurrence of complications and of side effects were monitored.

### 2.5 Statistical methods

Data analysis was performed using SPSS for Windows, version 23 (IBM Corporation). Normal distribution was tested for continuous variables. Non‐parametric tests were used in case of the absence of normal distribution. Data are presented as frequencies for categorical variables, and as median (interquartile range; IQR) in case of skewed distributions, or as mean (SD) in case of normal distributions for continuous variables. Chi‐squared test or Fisher's exact test (in case of small samples) was used to assess differences between categorical variables, whereas the Mann‐Whitney *U* test and Kruskal‐Wallis test were used for continuous variables. All statistical tests were two‐sided, with a significance level of 0.05.

## 3 RESULTS

A total of 86 patients diagnosed with gastroparesis (71% female, age range 20‐87 years [mean 55.8 years]) were seen between 2008 and 2016, and their data were analyzed. Idiopathic gastroparesis was the most common type of gastroparesis (n = 33, 38.3%), followed by diabetic gastroparesis (n = 23, 26.7%), postsurgical gastroparesis (n = 23, 26.7%), and generalized motility disorder (including patients with Ehlers‐Danlos syndrome) (n = 7, 8.1%). Baseline characteristics, etiologies per treatment group, and results of scintigraphy are shown in Table 1.

**Table 1:** Baseline characteristics of patients with gastroparesis

Patients with gastroparesis	Total n = 86 Median (IQR)	Group I (n = 50) DP only	Group II (n = 36) DP + GR	*P*‐value Group I vs Group II	Group III (n = 17) DP + GR responders	Group IV (n = 19) DP + GR non‐responders (PEG‐J)	*P*‐value Group III vs Group IV
Demographic parameters							
Age	60.0 (42.8‐67.0)	62.5 (49.5‐69.5)	54.00 (37.3‐62.0)	**0.008**	43.0 (31.0‐62.0)	56.0 (38.0‐62.0)	0.428
Gender (female, %)	71%	70%	72.2%	0.824	70.6%	73.7%	0.838
Etiology	N (%)	N (%)	N (%)	0.491	N (%)	N (%)	0.853
Idiopathic	33 (38.3)	16 (32)	17 (47.2)		7 (41.2)	10 (52.6)	
Diabetic	23 (26.7)	17 (34)	6 (16.7)		4 (23.5)	2 (10.5)	
Postsurgical	23 (26.7)	15 (30)	8 (22.2)		4 (23.5)	4 (21.1)	
Generalized GI motility disorder	7 (8.1)	2 (4)	5 (13.9)		2 (11.8)	3 (15.7)	
Gastric emptying parameters							
T50 solid (min; normal 64‐103 min)	134.0 (110.0‐177.0)	129.0 (108.5‐180.5)	139.0 (122.8‐174.8)	0.410	145.0 (129.0‐170.0)	129.0 (103.0‐195.0)	0.337
Stasis 60 min (%; normal 53%‐79%)	83.5 (72.0‐90.0)	81.5 (69.3‐87.8)	87.0 (77.3‐90.8)	0.109	86.0 (76.0‐87.5)	90.0 (82.0‐93.0)	0.087
Stasis 120 min (%; normal 16%‐37%)	54.5 (45.0‐73.0)	52.0 (45.0‐72.5)	59.5 (51.0‐73.0)	0.389	59.0 (51.0‐69.0)	61.0 (37.0‐73.0)	0.876
Stasis 240 min (%; normal 0%‐4%)	10.5 (1.0‐27.3)	10.0 (9.0‐28.0)	13.0 (5.0‐27.0)	0.407	14.5 (6.5‐27.3)	11.0 (1.0‐27.0)	0.553

DP, diet and prokinetics; GR, Gastric Rest; PEG‐J, Percutaneous Endoscopic Gastrostomy with jejunal extension.

Bold indicates statistical significance.

Groups were defined as follows: group I consists of patients that only needed DP (responders to DP, 50 patients). Group II consists of non‐responders to DP (decompensated gastroparesis), who received GR (36 patients). Group III consists of responders to DP + GR (17 patients), and Group IV of patients that did not respond to DP and GR and therefore received PEG‐J (19 patients).

Before treatment (6‐12 months), 31 patients had a stabile weight (36%), 27 patients (31.4%) had <10 kg weight loss, and 28 patients (32.6%) had ≥10 kg weight loss. No relation was observed between treatment (symptom as well as weight) response and gastric emptying parameters (data not shown).

### 3.1 Drug use

Of the 86 patients, 12 patients used opioids (only in three cases, it was apparent that opioid use was an etiological factor in gastroparesis due to the temporal relation between initiating opioids and gastroparesis symptom development). A total of seven patients used centrally acting agents (such as amitriptyline, escitalopram, sertraline, and paroxetine) next to GR or PEG‐J.

### 3.2 Endoscopic findings

Endoscopic findings consisted of diaphragmatic hernia (n = 8), mild gastritis (n = 5), mild erosions (n = 3), GAVE (n = 2), insufficient cardia (n = 2), Barrett esophagus (n = 1), candida esophagitis (n = 1), small ulcus (n = 1), and fundic gland polyps (n = 1).

### 3.3 Treatment results

#### 3.3.1 Diet and Prokinetics (DP)

All 86 patients were treated with diet and prokinetics.

##### Outcomes of DP

**Figure 1:**
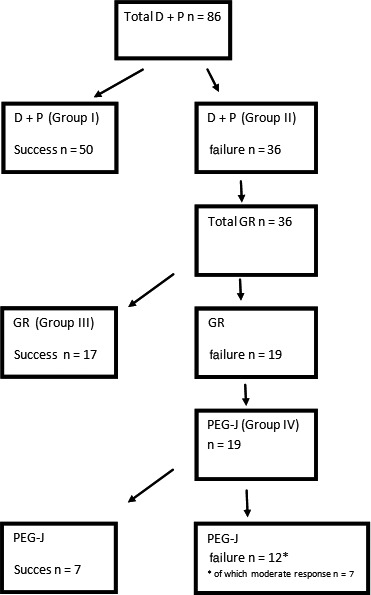
Treatment algorithm

Of the 86 patients, 50 patients (58%, group I) were symptom responders and did not require further therapeutic interventions (Figure 1). Symptom responders showed no significant change in weight (0.3%, *P* = 0.719), whereas non‐responders lost a significant amount of weight (6.2 kg, 8.7%, *P* = 0.006; Table 2) during the treatment phase. The weight change after treatment between symptom responders and non‐responders was significantly different (*P* = 0.005). Non‐responders to DP were significantly younger than responders (*P* = 0.008). The differences in scintigraphic parameters were non‐significant.

**Table 2:** Objective treatment outcomes in relation to subjective response

	N (total n = 86)	Mean weight (kg) before (SD)	Mean weight (kg) after (SD)	Mean weight change (kg) (%)	*P*‐value (pre‐ vs post‐treatment)
Diet & prokinetics (n = 86)	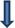				
Symptom responders	50	72.3 (14.8)	72.5 (14.9)	+0.2 kg (+0.3%)	0.719
Non‐responders	36	73.5 (15.4)	68.4 (13.1)	−6.1 kg (−8.3%)	**0.006**
Gastric Rest (n = 36) 3 months	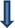				
Symptom responders	17	69.5 (13.5)	72.1 (14.7)	2.5 kg (3.6%)	**0.018**
Non‐responders	19	65.6 (13.2)	67.8 (15.23)	2.1 kg (3.3%)	**0.027**
PEG‐J (n = 19) 6 months	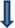				
All	19	64.9 (14.6)	69.9 (16.0)	5.1 kg (7.9%)	**0.002**
Responders 1‐2	7^a^	62.3 (10.7)	70.3 (13.6)	8.1 kg(12.9%)	**0.026**
Non‐responders (GPA 3‐6)	11^a^	64.7 (16.4)	68.1 (17.8)	3.5 kg (5.4%)	**<0.001**

a Outcomes were missing for one patient.

Bold indicates statistical significance.

##### Complications of DP

In total, four patients experienced side effects of the prokinetic medication. These included vertigo in one patient after domperidone (normal ECG/QT interval). One patient reported palpitations after use of metoclopramide (normal ECG/holter). After using erythromycin, one patient reported nausea, and one patient reported palpitations and stomach ache.

#### 3.3.2 Gastric Rest (GR)

A total of 36 patients (non‐responders to DP) were treated with three months of gastric rest by nasoduodenal tube feeding after insufficient response with DP. Enteral tube feeding was well tolerated by all patients. Only in case of complaints, tube position was checked.

##### Outcomes of GR

A total of 17 patients (47%, group III) responded well to GR: These patients were able to discontinue enteral feeding after three months and successfully re‐introduced oral intake (in a slow and stepwise manner, gradually increasing calories as tolerated). Oral intake was combined with medication such as prokinetics (erythromycin, domperidone, and metoclopramide) in 65% of patients. Objective weight gain in GR symptom responders was 2.5 kg (*P* = 0.018 compared to pretreatment; Table 2).

The other 19 patients (53%, group IV) were non‐responders with respect to not being able to discontinue enteral feeding and completely resume oral feeding. These 19 patients also had a weight gain of on average 2.1 kg (*P* = 0.027) after 3 months of GR Weight change and weight before and after treatment were not statistically different amongst responders and non‐responders to GR (resp. *P* = 0.433 and *P* = 0.531).

##### Complications of GR

In the 3 months of GR, complications included occlusion of the nasoduodenal tube in three patients (8%) and nasopharyngeal irritation in two patients (5.5%), resulting in tube replacement.

#### 3.3.3 Percutaneous Endoscopic Gastrostomy with jejunal extension (PEG‐J)

The 19 patients (22% of the total of 86 patients, group IV) with insufficient symptomatic response after 3 months GR continued treatment with enteral feeding through PEG‐J. PEG‐J placement was successful in all patients. An initial weight loss of mean 3 kg (range 0‐13 kg) was observed after GR was stopped.

##### Outcome of PEG‐J

###### Subjective outcome

Marked relief was reported by seven patients (36.8%, GPA 2), moderate relief by 7 (36.8% GPA 3), and slight relief in two patients (10.5%, GPA 4). Three patients reported minor or no relief (15.8%, GPA 5).

##### Objective outcome

A significant weight gain of (mean) 5.1 kg (range −5 to + 21 kg, *P* = 0.002) was observed within 6 months after PEG‐J placement. Symptom responders showed a more pronounced weight gain compared with non‐responders (Table 2), although weight and weight change were not statistically different amongst responders and non‐responders for PEG‐J (resp. *P* = 0.150 and *P* = 0.454).

##### Intake

Of all patients receiving enteral nutrition, 16 (84%) were able to have some oral intake in addition to enteral tube feeding, while three patients could not eat or drink anything (16%).

##### Manometric data (subjective responders vs non‐responders)

Data were eligible for analysis in 17 of the 19 patients (no measurement performed in two patients). No significant differences were observed in manometry data (antral amplitude, antral motility index, and presence of phase III contractions) between responders (n = 7) and non‐responders (n = 12) to PEG‐J, albeit this might be due to a type II statistical error.

##### Complications of PEG‐J

Most frequent complication was luxation of the jejunal extension to the stomach (six patients [32%], 1‐8 times), occurring after a few months of use, which required placement of a new extension. Other complications were peristomal infection (in two patients [10.5%] within 30 days, in three patients [16%] after 30 days, easily treated with oral antibiotics) and buried bumper (three patients, 16%, after approximately one year which lead to replacement).

##### Long‐term outcomes of PEG‐J

Only three patients (16%) of the 19 patients treated with PEG‐J were able to resume complete oral intake. The PEG‐J was removed after a mean of 11 months. In 84% of patients (n = 16), the PEG‐J is still in use, with a mean treatment time of 32 months. Besides PEG‐J feeding, 13/19 (68%) of patients needed additional treatment with prokinetics/antiemetic medication. No significant differences were seen in manometric or scintigraphic results of the patients that have been able to completely resume oral feeding compared to patients that still use PEG‐J.

## 4 DISCUSSION

This study describes the follow‐up of a cohort of tertiary referral gastroparesis patients treated with a stepwise approach of standardized interventions. Considering symptom response, diet and prokinetic treatment was effective in relieving symptoms in 58% of patients. In patients refractory to diet and prokinetic treatment, gastric rest with enteral nutrition effectively improved symptoms in an additional 20% (17/86) of patients. In severely decompensated gastroparesis patients who failed to respond to the two previous treatment steps, PEG‐J was an efficacious treatment entity in an additional 8% (7/86) of patients. Taken together, our approach resulted in 86% efficacy when combining the results of the three stepwise interventions. In 12 of the 86 patients (14%), we did not achieve an adequate symptom response: seven patients only had a moderate symptom response, and five patients had little or no response at all. Another important finding from our study is that the nutritional interventions (applied according to our stepwise approach), resulted in significant weight gain, independent of symptom response.

The overall response to diet and medication (step 1) was 58%, the response to gastric rest with enteral nutrition (step 2) was 47%, and the response to PEG‐J (step 3) was 37%. The question arises why some patients with gastroparesis respond or not respond to the subsequent steps of treatment and which factors contribute to treatment success or failure.

For the first step, medication and diet, we systematically evaluated whether the available prokinetics (erythromycin, domperidone, and metoclopramide) had been tried for a period of at least four weeks per medication. The *non‐responders* to diet and prokinetics were characterized by a significantly younger age. Previously, higher age (50 years and older) was shown to have better symptom response in one study.^38^ Scintigraphic parameters were not significantly different between responders and non‐responders.

For the second step with gastric rest and enteral tube feeding, no significant differences were observed between responders and non‐responders, neither in clinical parameters, nor in cause of gastroparesis, nor in results of scintigraphy.

For the third step with PEG‐J, neither clinical parameters, nor results of scintigraphy, nor antroduodenal manometry data were significantly different between responders and non‐responders to PEG‐J intervention, albeit that this was a fairly small group in numbers. Thus, the response to treatment either in terms of weight gain or symptoms was not correlated to data of scintigraphy or motility. It therefore appears that gastric motor function characteristics do not affect the response to treatment. This observation is largely in line with the consistent observation that gastric emptying studies (scintigraphy as well as wireless motility capsule) show poor correlation to symptoms of gastroparesis.^39^, ^40^, ^41^, ^42^, ^43^, ^44^

A potential factor that we did not systematically take into account is the presence of psychiatric comorbidities, especially depression or anxiety disorder and whether patients underwent any specific treatment for these conditions that could have influenced treatment response to nutritional interventions. Literature data indicate that psychiatric comorbidities are frequent in patients with gastroparesis: combined anxiety/ depression in 24%, severe anxiety in 12%, depression in 23%, and somatization in 50%.^45^ Acute anxiety and stress are known for their profound influences on gastric motor function, in particular on gastric accommodation.^46^ In addition, psychoactive medication has been suggested to influence gastric motility 47 although literature findings are inconsistent.^48^ This appears relevant as these drugs are often prescribed for relief of abdominal symptoms in patients with gastroparesis even in the absence of apparent psychopathology. It is noteworthy that the NORIG study has recently shown that nortriptyline is not effective for symptom relief in gastroparesis patients.^49^ Here, we argue in favor of routine assessment of psychiatric comorbidities in patients with severe gastroparesis and taking into account psychiatric comorbidities when evaluating therapeutic efficacy of treatment modalities.

Based on literature data, approximately 30% of gastroparesis patients progress to grade III (decompensated) gastroparesis, of which the majority are eventually in need of long‐term enteral tube feeding.^50^, ^51^ In our hands, only 19 of 86 patients (22%) progressed to grade III. We have chosen for placement of PEG‐J with fixation of the tip of the tube in the distal duodenum or proximal jejunum. Alternatives procedures include jejunostomies either placed via surgery or radiology or endoscopy.^52^ The surgical approach is known for higher complication rates 53, 54 and experience with (direct) PEJ (Percutaneous Endoscopic Jejunostomy), and percutaneous radiologic jejunostomy (PRJ) in gastroparesis is limited.^55^, ^56^, ^57^ In a study assessing the effect of surgical jejunostomy in diabetic gastroparesis, symptom relief was seen in 39% and improved nutritional status in 56%.^52^ No data on the efficacy of nasojejunal feeding or the effect of dietary treatment on weight in gastroparesis patients were published earlier. Our choice in favor of the PEG‐J procedure with duodenal extension and fixation is based on local expertise of our PEG team and outpatient PEG support.

Several other therapeutic entities are available. Botulinum toxin injections were performed at our center in the period that these patients were included. Some patients had been treated with botulinum toxin (22/86). However, botulinum toxin therapy was not the primary focus of our analysis. In addition, this therapy is not recommended by current guidelines due to questionable response when compared to placebo, and therefore, we discourage the use of this intervention and have abandoned this practice completely as we have done so in our center in 2017.

One of the non‐responders received gastric pacing (abroad), and one patient had a surgical pylorotomy. G‐POEM as a therapeutic entity has only been offered recently at our center (2018), so no patients subject to the current analysis have received such treatment.

A procedural success rate of 100% was found in our study for both gastroduodenal tube placement and PEG‐J, which is comparable to literature rates.^34^, ^56^, ^57^ In 3 months of nasoduodenal feeding (GR), occlusion of the tube and nasal irritation occurred at relatively low rates (resp. 8% and 5.5%). Patients were able to accept the NJ fairly well, because in advance, they agreed to a 3‐month period. In addition, patients were informed that PEG(‐J) placement is a procedure which is generally associated with more risks and complications that an NJ tube. In addition, the NJ tube is fairly thin, which may have contributed to good tolerability.

Dislocation is the major disadvantage of PEG‐J and occurred in 32% of patients. These rates are in line with literature data, ranging from 25.2% to 55.9%.^34^, ^58^ No occlusion occurred, which is described in 3.5%‐35% of patients with PEG‐J.^34^, ^35^

Some limitations of our study should be mentioned. Firstly, it is a retrospective analysis of prospectively collected data. Over the eight‐year period, we did not systematically collect all patient‐reported outcome measures or systematically characterized psychological factors and psychiatric comorbidities. Secondly, we serve as tertiary referral center. Therefore, a small percentage (9%) of the scintigraphic data were collected in other centers with different protocols and normal values and were therefore not eligible for statistical comparison. We, however, believe these are reliable in diagnosing gastroparesis.

Antroduodenal manometry results were only taken into account with respect to antral contractility and the presence of phase III‐activity. Other parameters such as intestinal contractile activity, propagation of contractions, or presence of pathological motility patterns were not analyzed. Thirdly, the overall number of patients we included is substantial but is limited with respect to subgroup analyses. Due to our referral function, often a more pragmatic approach is followed with a trial of prokinetics in patients presenting with dyspepsia without prior testing of gastric emptying, which is a prerequisite to establish the diagnosis of gastroparesis. These patients are not tested for gastric emptying when showing a positive therapeutic response to prokinetics and could potentially have had gastroparesis rather than functional dyspepsia. Otherwise, our sample size would have been larger.

Finally, AEs were reported in some of our patients after using prokinetics. In all other patients, no specific AEs were reported in medical history. We cannot therefore rule out that AEs have been underreported.

A strength of our study is that it is the first study reporting on the stepwise therapeutic approach of gastroparesis and on the role of gastric rest and of PEG‐J, with respect to objective as well as subjective treatment outcomes. Moreover, it is the largest study since Fontana in 1996 (including 26 patients receiving surgical jejunostomy), reporting on the effects of enteral tube feeding in gastroparesis.^17^, ^52^ Other available studies considering enteral feeding in gastroparesis (surgical or direct jejunostomy) included small populations of two, four, and twelve patients.^55^, ^56^, ^57^ Our analysis revealed only minor complications using PEG‐J (with replacement of the tube in 16% of patients), whereas with the surgical approach, 14/26 patients had one or more major complications (requiring hospitalization or surgery).^52^ With direct PEJ, the rate of complications was 18.2% (2/11), including volvulus and a jejunocolic fistula.^55^ Direct PEJ showed lower technical success (78.6%), whereas we had a 100% technical success rate. Only 56% of patients with surgical jejunostomy had improved nutritional status,^52^ whereas virtually all our patients showed significant weight gain with PEG‐J.

Our findings have implications for therapy in patients with severe gastroparesis. Firstly, 86% of the referred gastroparesis patients showed adequate clinical responses to our stepwise systematic approach. Secondly, nutritional interventions aiming to restore the nutritional status are crucial. We persisted in a full three‐month regime with gastric rest based on expert opinion. Gastric rest may lead to decompression of the stomach, to provide time for recovery of motility and a new set point that allows the successful reintroduction of oral intake. Others may argue that oral intake may be continued while on enteral nutrition, because partial gastric rest may already prove to be sufficient. In this respect, evidence in favor for stricter or more liberal gastric rest is lacking.

## 5 CONCLUSIONS

Following a stepwise treatment approach in gastroparesis, adequate symptom response was reached in 86% of patients, with weight gain in virtually all patients, independent of the symptom response. Diet and prokinetic treatment was effective in 58% of patients with gastroparesis. In decompensated patients, refractory to diet and prokinetic treatment, gastric rest with nasoduodenal tube feeding was effective in 47% and, in patients who failed all previous treatments, PEG‐J appeared to be an efficacious alternative in 37% of patients. Despite the limited number of therapeutic options, a rigorous stepwise approach resulted in an acceptable success rate in tertiary referred patients with gastroparesis.

## DISCLOSURES

No competing interests declared.

## AUTHOR CONTRIBUTIONS

DS performed the research; DS analyzed the data and wrote the paper; FS partially analyzed the data, AM and DK designed the research study and revised the paper. FS, JK, LG, RR and JC revised the paper.
